# Acute glycaemic response of orange juice consumption with breakfast in individuals with type 2 diabetes: a randomized cross-over trial

**DOI:** 10.1038/s41387-025-00385-8

**Published:** 2025-07-09

**Authors:** Kenneth Verboven, Lisa Van Ryckeghem, Ralf Schweiggert, Christof B. Steingass, Tin Gojevic, Carrie H. S. Ruxton, Dominique Hansen

**Affiliations:** 1 BIOMED Biomedical Research Institute, Hasselt University, Diepenbeek, Belgium; 2 REVAL Rehabilitation Research Center, Hasselt University, Hasselt, Belgium; 3 Chair of Analysis and Technology of Plant-based Foods, Department of Beverage Research, Geisenheim University, Geisenheim, Germany; 4 Nutrition Communications, Cupar, UK; 5 Heart Center Hasselt, Jessa Hospital Hasselt, Hasselt, Belgium

**Keywords:** Type 2 diabetes, Nutrition

## Abstract

**Background/Objectives:**

Sugar-sweetened beverages are associated with an increased risk of obesity and type 2 diabetes (T2DM) and show clear differential metabolic responses compared with 100% fruit juice, which is unsweetened by law. This study investigated whether the postprandial glycaemic response following a standardized breakfast differed when accompanied by 100% orange juice, equivalent whole orange, or a sugar-sweetened control beverage in individuals with well-controlled T2DM.

**Subjects/Methods:**

Fifteen individuals with T2DM (60 ± 6 y; BMI 28.7 ± 5.0 kg/m², HbA1C 49 ± 3 mmol/mol (6.6 ± 0.3%)) participated in this randomized cross-over trial. They consumed a standardized breakfast served with either 250 mL of 100% orange juice, a sugar-sweetened orange-flavoured beverage or whole orange pieces with identical total sugar content. Postprandial glycaemic and insulinaemic responses were checked during 4 h.

**Results:**

Following a single intake, no significant differences were found in acute glucose or insulin responses (expressed as total or incremental area under the curve or peak values; *p*_treatment_ > 0.05, respectively) when either whole orange pieces, orange juice or a sugar-sweetened control beverage were consumed with a standard high carbohydrate meal. Capillary glucose responses did not differ between conditions (*p*_treatment_ > 0.05).

**Conclusion:**

Acute glycaemic control in individuals with well-controlled T2DM is not significantly influenced by serving orange juice, whole orange pieces or a sugar-sweetened beverage with a standard high-carbohydrate meal.

## Introduction

Recent epidemiological data show increased prevalence rates of type 2 diabetes mellitus (T2DM) worldwide, accompanied by a raised incidence of diabetic complications [1]. To improve diabetes care and to diminish the risk of diabetes-related complications and all-cause mortality, healthy lifestyle behaviours, including physical activity and healthier diets, should be promoted for these patients [2].

Sugar-sweetened beverages are frequently consumed in middle-to-high income societies but have been associated with an increased risk of T2DM [3]. They are not recommended for people with existing T2DM [4]. The evidence for 100% fruit juice, a source of natural fruit-based sugars which are classified as ‘free sugars’, shows a clear differential metabolic response compared with sugar-sweetened beverages in human experimental studies [5, 6]. Indeed, a meta-analysis of randomised controlled trials suggested that long-term consumption of 100% orange juice does not have an adverse impact on indices of glycaemic control or insulin sensitivity in study populations varying in health states [7]. This may result from the fact that citrus fruits are an important source of dietary flavonoids, a group of polyphenols which were originally classified as generic antioxidants [8]. Of particular interest is hesperidin, a naturally occurring glycoside of the flavanone hesperetin, found abundantly in citrus fruits. The consumption of this compound has been associated with a plethora of putative pharmacologic properties, including reports about blood glucose lowering properties [9]. In healthy individuals, hesperidin significantly slowed intestinal glucose uptake, thereby altering post-prandial glycaemic control [10].

However, the literature on consumption of 100% fruit juice in individuals with T2DM is highly variable, with a few studies reporting acute negative glycaemic effects when consumed with or without a meal [11, 12]. Dietary guidelines for people with T2DM are also inconsistent with some recommending that whole fruits be consumed instead of fruit juice, while others advocate modest servings of fruit juice up to 150 mL per day [4]. Hence, it is unclear whether people with T2DM may safely consume 100% fruit juice with a meal in the same way they would a matched serving of whole fruits.

The present study aimed to test whether the postprandial glycaemic response following a standardised high carbohydrate breakfast differed when accompanied by 100% orange juice, an equivalent of orange fruit pieces, or a sugar-sweetened control beverage, all supplying an identical amount of total sugar. The study employed a randomised cross-over design in individuals with well-controlled T2DM. It was hypothesised, based on the available evidence in populations without diabetes, that more favourable effects on glycaemic control would be seen for whole orange, while less favourable effects would be seen for the sugar-sweetened beverage, with the 100% orange juice somewhere in-between. The hesperidin naturally present in the orange fruit and the 100% orange juice could also be hypothesised to have a favourable effect on glycaemic control.

## Materials and methods

### Subjects and randomisation

A total of 15 individuals with T2DM (including 5 women and 10 men, age 60 ± 6 year, body mass index (BMI) 28.7 ± 5.0 kg/m², HbA1c 49 ± 3 mmol/mol (6.6 ± 0.3%)) on blood glucose-lowering treatment (Table 1) were recruited for this prospective randomised cross-over trial via social media advertisement and by general practitioners’ referral. Patients were included based on clinical diagnosis or glycated haemoglobin (HbA1c) ≥ 48 mmol/mol (6.5%), age 25–75 years and a BMI 22–35 kg/m². Exclusion criteria comprised the use of exogenous insulin therapy, HbA1c ≥ 69 mmol/mol (8.5%), regular smokers (>1 cigarette per day), pregnancy or the presence of established cardiovascular, neurological, renal disease or cancer. Participants unwilling to consume any of the standard breakfast elements (either food or drinks) were excluded.

**Table 1:** Patient characteristics.

Gender (male:female)	10 : 5
Age (years)	60 ± 6
Length (m)	1.72 ± 0.10
Weight (kg)	85.8 ± 16.8
BMI (kg/m²)	28.7 ± 5.0
HbA1c (%)	6.6 ± 0.3
HbA1c (mmol/mol)	49 ± 3
HOMA-IR	3.6 ± 2.0
Metformin (n)	14
Sulfonylurea (n)	3
DPP4-inhibitor (n)	2
SGLT2 inhibitor (n)	3
GLP-1 receptor antagonist (n)	2
Statins (n)	12
Fibrates (n)	1
Antiplatelet (n)	3
Calcium antagonist (n)	5
ACE inhibitor (n)	4
Angiotensin-2 receptor antagonist (n)	2
Diuretics (n)	2
Beta blocker (n)	4
Other (n)	9

Data are means ± SD. Assessment of insulin resistance.

*BMI* body mass index, *HOMA-IR* Homeostasis Model Assessment of Insulin Resistance, *HbA1c* blood glycated hemoglobin.

Eligible patients with T2DM were randomised to a varied chronology of exposure conditions by sealed envelope, determined by an independent researcher not involved in the intervention or assessments. Participants were able to choose between two energy/carbohydrate-matched standard breakfast options: (a) ham version: 526 kCal, 79.5 g carbohydrates, 5.6 g fibres, 13.1 g fat and 10.1 g protein per serving (respectively); or (b) cheese version: 489 kCal, 81.2 g carbohydrates, 5.6 g fibres, 7.9 g fat and 3.9 g protein per serving (respectively). The breakfast of choice was served with either 250 mL of 100% orange juice (OJ-H), 250 mL sugar-sweetened orange-flavoured beverage (SOB) or whole orange pieces with peel removed (WOP), supplying circa 21.5–22 g total sugar. Due to natural variability, the sugar content of the fruit pieces was monitored by 16 separate analyses during the study. The total sugar content varied from 16.8 to 27.8 g total sugar per 250 g portion of fruit pieces. Similarly, the hesperidin dose fed via fruit pieces varied from 38.5 to 294.8 mg/250 g (mean 160 mg/250 g). The OJ-H was sourced from a single batch of concentrate, as described previously [13], having a highly uniform composition throughout the study. The OJ-H contained 134.3 mg hesperidin per 250 mL (537 mg/L), thus being at the higher end of the reference range under the orange juice Code of Practice (European Fruit Juice Association, May 13th 2024). The detailed composition of the test foods is described in Table 2. The development of the SOB has also been described previously [13]. Each of the breakfast conditions provided ~20% of the daily reference energy intake (8 372 kJ or 2000 kcal), followed by a standard snack (30 g of salted potato chips) 3 h later. *Ad libitum* water intake was permitted during the visits. Compositional details of the different orange conditions are provided in Table 2. No side effects related to the interventions were reported throughout the study period. The study was performed in accordance with the standards set by the latest revision (2013) of the Declaration of Helsinki and was approved by the local ethics committee (Hasselt University, Belgium). All participants gave written informed consent after careful explanation about the nature and risks of the experimental procedures of the study (NCT04412798).

**Table 2:** Composition of orange juice rich in hesperidin (OJ-H), sweetened orange-flavoured beverage (SOB), and whole orange pieces with removed peel (WOP).

		Orange Juice	Sugar-sweetened	Whole orange		
	Method		beverage	pieces		
		Mean	Mean	Mean ± SD	Min	Max
Total soluble solids (°Brix)	refractometry	11.7	9.1	12.0 ± 1.0	9.8	13.3
Glucose (g/L)^a^	enzymatic assay	18.3	24.9	21.5 ± 4.6	13.0	29.0
Fructose (g/L)^a^	enzymatic assay	22.2	28.4	23.2 ± 4.1	16.0	30.0
Sucrose (g/L)^a^	enzymatic assay	46.2	32.5	43.0 ± 7.8	30.5	57.2
Total sugars (g/L)^a^	calculated	86.7	85.5	87.8 ± 13.1	67.0	111.2
Glucose : fructose ratio	calculated	0.82	0.88	0.92 ± 0.04	0.82	1.00
Total dietary fibre (g/L)^a^	estimated based on literature	7.3	-	10.0	n.d.	n.d.
Ascorbic acid (mg/L)^a^	iodometry	275	4	405 ± 65	297	533
Citric acid (g/L)^a^	enzymatic assay	6.99	6.29	7.80 ± 2.46	4.19	13.95
Total phenols (mg/L)^a^	Folin-Ciocalteu assay (as (+)-catechin)	905	39	842		
Hesperidin (mg/L)^a^	HPLC-DAD	537	-	638 ± 294	154	1179
Potassium (mg/L)^a^	AAS	2446	-	1874 ± 328	1415	2617
Extract (g/L)^a^	calculated	122.7	94.9	112.4 ± 14.6	89.1	140.2
Sugar-free extract (g/L)^a^	calculated	36.0	9.1	24.6 ± 3.4	18.8	30.1

Data are expressed as means (juice / beverage) or as means ± standard deviations (SD) and min./max. values of whole orange pieces (derived from *n* = 16 individual servings).

Composition of whole orange pieces (relative to kg instead of L) determined on freshly collected whole-tissue mixtures (stored frozen until analyses).

^a^expressions in g/L or mg/L are valid for orange juice (OJ-H and SOB), expression in g/kg or mg/kg for whole orange pieces (WOP).

### Clinical characterisation and blood profiles

Patients arrived at 8:00 AM at the research facilities after an overnight fast. Height (stadiometer) and weight (Polar Balance, Polar, Finland) were determined. After obtaining fasted blood samples, the standard breakfasts were consumed. Serial blood samples were taken during a 4-h timeframe with a sampling frequency of 10 min in the first two hours and 20 min in the last two hours. At standardised timepoints, capillary blood glucose levels were checked from the fingertip (Accu-Chek Aviva, Roche Diagnostics, Machelen, Belgium). Sodium fluoride and serum tubes were centrifuged at room temperature for 15 min at 1650 g, and plasma and serum were stored at −80 °C at the UBiLim biobank facilities until analysis. Serial plasma and serum samples were measured by the clinical laboratory (Jessa Hospital, Hasselt, Belgium) for glucose and insulin (Roche Cobas 8000; Roche Diagnostics International Ltd, Rotkreuz, Switzerland). Additionally, HbA1C was determined (Menarini HA-8180 HbA1C autoanalyzer; Menarini Diagnostics, Diegem, Belgium). All coefficients of variation for these assays were less than 5%. Insulin resistance (IR) was assessed via homeostasis assessment of IR (HOMA-IR), calculated as (fasting plasma glucose (mmol/L) x fasting serum insulin (mU/L))/22.5 [14]. Beta-cell function was estimated via HOMA- β (%), calculated as (20 x fasting serum insulin (µU/mL))/(fasting plasma glucose (mmol/L) – 3.5) [14]. Total area under the curve (tAUC) for glucose and insulin and incremental AUC (iAUC) for glucose were calculated by the trapezoid method in the postprandial phase. Peak glucose and insulin levels and time to peak were calculated for each condition.

### Statistical analysis

**Fig. 1:**
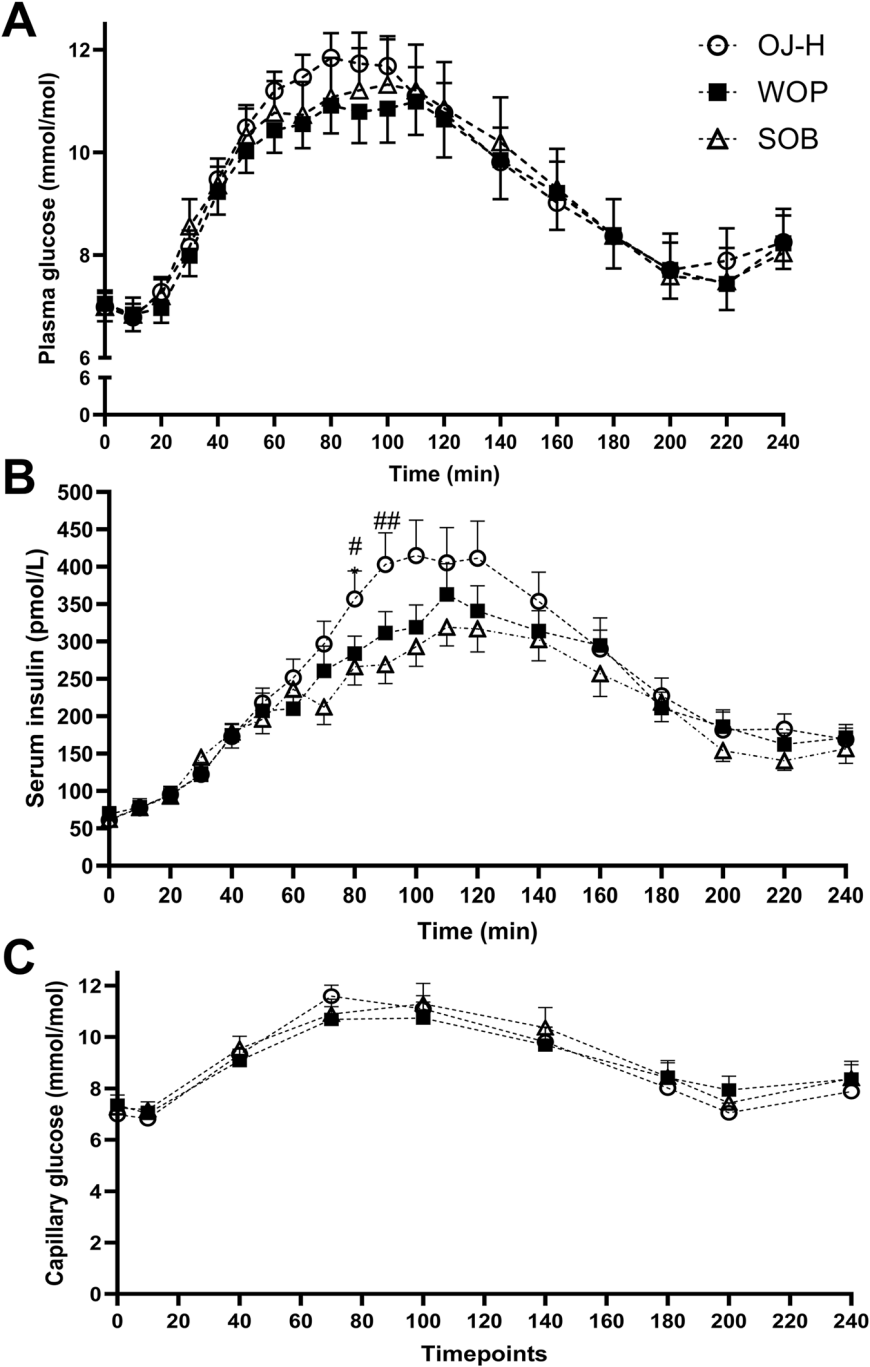
Glycaemic and insulin responses. Data represent means ± SEM (glucose: *n* = 15; insulin: *n* = 13). Plasma glucose (**A**), serum insulin (**B**) and capillary glucose (**C**) concentrations at rest and in the subsequent postprandial state following the consumption of three different fruit juices. * Significantly different between orange juice and fresh orange pieces (*P < 0.05). # Significantly different between orange juice and sugar-sweetened orange-flavoured beverage (#*P* < 0.05; ##*P* < 0 .01).

Data are presented as means ± SD (Table 1) or SEM (Fig. 1). Shapiro-Wilk test indicated no normal distribution of data. To compare blood responses (interval data) between different experimental conditions, Friedman tests (three related samples) were performed. In case of a significant main effect, *post hoc* Wilcoxon signed ranks tests (for two related samples) were performed corrected for multiple testing (Bonferroni). Total area under the curve (tAUC) and incremental area under the curve (iAUC) for glucose and insulin were calculated by the trapezoid method in postprandial phase. Statistical significance was set at *P* < 0.05 (two-tailed) for main effects and *P* < 0.017 (two-tailed) for two related conditions. SPSS 29 for Windows was used for statistical analyses (IBM Corporation, Armonk, NY, USA).

## Results

### Plasma glucose

For venous glycaemic responses, neither tAUC_glucose_, nor iAUC_glucose_ differed between conditions (P_treatment_> 0.1 for all postprandial phases (0–60 min, 0–120 min, 0–180 min and 0–240 min, respectively)) (Supplemental Table 1). Postprandial peak glucose concentrations and time to reach those were similar between conditions (P_treatment_ = 0.119 and 0.595, respectively) (Fig. 1A). Capillary glucose responses did not differ between conditions either (Fig. 1C). Secondary analyses for glycaemic responses based on sex, HOMA-β or BMI categories revealed no differential responses for tAUC_glucose_, iAUC_glucose_, peak glucose or time to peak glucose (data not shown).

### Serum insulin

Postprandial insulinaemia, either expressed as tAUC_insulin_ or iAUC_insulin_, did not differ between conditions (P_treatment_>0.05 for all postprandial phases (0–60 min, 0–120 min, 0–180 min and 0–240 min, respectively)) (Supplemental Table 1). Of interest, peak insulin tended to be different between experimental visits (P_treatment_ = 0.056), while time to reach the peak was similar (P_treatment_ = 0.646) (Fig. 1B). Based on secondary analyses for postprandial insulin responses, female individuals with T2DM tended to show a differential response between conditions when expressed as tAUC_insulin_ (P_treatment_ = 0.022), iAUC_insulin_ (P_treatment_ = 0.015) or peak insulin (P_treatment_ = 0.022). Individuals with normal to mild impaired beta-cell function (HOMA-β > 70%) showed a differential response between conditions when expressed as iAUC_insulin_ (P_treatment_ = 0.009) or peak insulin (P_treatment_ = 0.009), while those with moderate impairment (HOMA-β < 70%) showed no differential responses between conditions. Considering BMI categories, overweight individuals with T2DM tended to show a differential response between conditions, when expressed as tAUC_insulin_ (P_treatment_ = 0.041), iAUC_insulin_ (P_treatment_ = 0.022) or peak insulin (P_treatment_ = 0.007) (data not shown).

## Discussion

Evidence-based, realistic dietary advice is needed for people with T2DM to manage and improve their condition and overall cardiometabolic health. Increasing fruit and vegetables is one aspect of this, as it is for the general healthy population, but the role of 100% fruit juice (i.e., no added sugars, as described in the Reference Guidelines of the European Fruit Juice Association’s Code of Practice) requires further clarification, especially for populations with diabetes. Contrary to expectations, the current study found no significant differences in acute glucose or insulin responses when either whole orange pieces, orange juice or a sugar-sweetened control beverage identical in total sugar content were consumed with a standard meal rich in carbohydrates. One reason for this could be that the carbohydrate load of the three test options (~22 g) contributed to a lesser extent to the observed glycaemic responses when compared with the carbohydrate load of the breakfast meal (~80 g). Intriguingly, it appears that certain nutritional aspects of whole orange pieces (containing hesperidin and fibre) and 100% orange juice (containing hesperidin) were insufficient to influence the overall glycaemic response when consumed with a carbohydrate-rich meal. In this context, the inherently high variability of the composition of fruit pieces as compared to the homogeneous composition of the orange juices sourced from a single batch should be highlighted (Table 2).

As the potential health benefits of dietary polyphenols is a growing field of interest in nutritional science, previous studies in populations without T2DM have found that experimental hesperidin, which remains unmodified until it reaches the colon, decreases postprandial glycaemia via GLUT-2 inhibition [10]. GLUT-2 transporters at the basolateral membrane of the enterocytes (i.e., small intestine) make passive diffusion of glucose/fructose/galactose possible into the bloodstream. However, in insulin-resistant individuals with obesity, GLUT-2 is known to accumulate in the apical membrane, allowing glucose to passively diffuse into the bloodstream [15]. Although the orange juice and whole orange pieces in the current study were consumed in an acute setting, our findings appear to be in line with previous study results which showed that individuals with obesity and/or a reduced insulin sensitivity, as is typically the case in T2DM, hamper any hesperidin-related glucose lowering effects [16]. Moreover, a 12-week intervention with daily 100% orange juice versus a sugar-matched orange-coloured drink in overweight and insulin-resistant men reported no alterations in carbohydrate or lipid metabolism [17]. Since beta-cell functional capacity (often expressed by the proxy HOMA-β [14]) is a known parameter (at least partly) determining acute glycaemic control, being related to metabolic clearance rates of endogenous insulin or the acute induction of insulin resistance [18], we observed a heterogenous range (i.e., HOMA-β 28–132%) among the individuals included. Therefore, it is tempting to speculate that this variation in beta-cell function might have blunted the current studied glycaemic responses. Unfortunately, the current study lacked statistical power to perform secondary analyses based on BMI or HOMA- β.

Interestingly, as no differences in glycaemic control between experimental conditions were found in our acute study, the current data may indicate the importance of colonic modification (as reviewed by Mas-Capdevila et al. [19]). Indeed, the cardiometabolic health benefits of citrus flavonoids, like hesperidin, on postprandial carbohydrate metabolism may not solely rely on small intestinal function. Factors like inhibitory effects on intestinal α-glucosidase, gut microbiota composition, substrate utilisation or mitochondrial function may impact these effects in metabolically compromised individuals [20–22].

Although our study did not investigate the chronic effects of fruit juice consumption, we note that the recommendations of the European Association for the Study of Diabetes which state that “the evidence for benefits for 100% fruit juice appears restricted to levels of intake obtainable from a single piece of fruit (≤150 ml)”. In accordance with this statement, our results suggest that a single serving of orange pieces or 100% orange juice with a meal does not have a detrimental effect on glycaemic control in people with T2DM. Nevertheless, fruit juices are certainly sugar-containing beverages and, according to numerous dietary recommendations [4], they should only be consumed occasionally. Unlike fruit juices, sugar-sweetened beverages do not contain similar vitamins, minerals and natural bioactive compounds as fruits. Therefore, sugar-sweetened beverages remain inadvisable for people with T2DM, despite the findings of this study.

The main limitations of the current intervention include the small sample size, limiting the detection of small differences in (potential metabolically important) glycaemic outcomes, and the partial blinding of the participants. However, experimental visits were randomised so it is unlikely that a single postprandial intervention would change the response to the next experimental visit (minimal break of 5 days between experimental visits). Strengths of the intervention include the measurement of glucose using both venous and capillary methods, the standardisation of the orange juice and the measurement of hesperidin in both the orange juice and all 16 individual batches of the served whole orange pieces. Future studies should examine the long-term impact of eating fruit versus drinking fruit juice on glycaemic control and insulin sensitivity in people with T2DM, considering more robust or sensitive markers other than AUC, such as glycated haemoglobin.

In conclusion, our results indicated no differences in acute glycaemic control in individuals with well-controlled T2DM following a single serving of orange juice, whole orange pieces or a sugar-sweetened beverage with a standard high-carbohydrate meal. In addition, given the similarity of outcomes for the two orange conditions (containing hesperidin) and the sugar-sweetened beverage control (no hesperidin), a single dose of hesperidin had no acute impact on glycaemic control.

## Supplementary information

Supplemental Table 1 - Plasma glucose and serum insulin concentrations during different breakfast conditions

## Data Availability

The datasets generated during and/or analysed during the current study are available from the corresponding author on reasonable request.
